# Granulations and Differential Diagnosis of Cases Favorable and Cases Unfavorable

**Published:** 1921-04

**Authors:** Thomas P. Hinman


### GRANULATIONS AND DIFFERENTIAL DIAGNOSIS OF CASES FAVORABLE AND CASES UNFAVORABLE

BY THOMAS P. HINMAN, D.D.S.

The question of differential diagnosis between salvable teeth and teeth that should be extracted is one that has given the conscientious practitioner more real anxiety than any other problem in present-day practice, and it is the intention of this paper to make clear, if possible, some of the points in differential diagnosis as shown by the X-ray pictures.

When it has been determined that a pulpless tooth showing a rarefied subapical area is to be treated with the expectancy that this subapical area will return to normal, the following questions arise: (1) What is the condition of the apical cementum and how can this condition be determined? (2) What is the condition of the subapical bone and how may we determine this? (3) After determining that conditions are favorable for treatment, what methods should be used to bring about a condition favorable for restoration of the subapical bone, and may we expect a reproduction of the destroyed area? (4) In what length of time should we expect bone restoration? (5) Are teeth so treated no longer a menace to health? I will endeavor to answer these questions seriatim.

1. What is the condition of the apical cementum and how can this condition be determined?

First, the condition of the apical cementum may be determined by the careful examination of good, clear X-ray pictures, not the usual type so often seen in which a central incisor root is shown to be so long that it looks like a Lombardy poplar, but a picture that shows accurately the root length, the end cementum, the lamina dura and bone trabeculae. If an attempt is made to show the relation of the tooth to the peridental membrane, to the lamina dura, to the apical cementum, and to the subapical bone, it is extremely important to show the relation definitely without either foreshortening or extreme lengthening of the shadow picture. If a question arises as to the lack of definiteness of these areas, then the tooth should be photographed from three distinct angles. If it is the incisor, it should be photographed labio-lingually, focusing on the central portion of the tooth; then a picture should be made using the mesial angle as a focal point; and for the third one, the distal angle should be used as a focal point. It is sometimes astonishing how much information, which the single picture has not clearly revealed, can be obtained from the additional pictures.

If the apical cementum shows rarefaction, no amount of treatment will avail. On the other hand, if the apical cementum shows no rarefaction, treatment is indicated. If the apical cementum shows condensation, treatment is again indicated. In cases where we have condensation, or adventitious cementum, the sections of these root ends have shown that the majority of the foramina are closed; frequently only a single apical foramen is open, from which it would seem that nature has endeavored to close off infection. This condition of the apical cementum is clearly shown in correctly taken; accurate X-ray pictures.

2. What is the condition of the subapical bone and how may we determine this?

The condition of the subapical bone should be clearly shown in the X-ray picture and is readily determined by a correct reading. This condition is of great importance in determining the question of treatment, for if this bone is infiltrated and there is no definite limited area, we have a condition of bone infiltration known as rarefying osteitis. The area of destroyed bone is usually very much larger than is shown by the X-ray picture. This has been determined by frequently cutting down on these areas and examining them.

Cases of this type do not yield readily to treatment, and where the area is of considerable extent treatment is contraindicated even if the apical cementum be in very good condition. It has been noted in these cases that the resistance of the patient is, as a rule, very low, and nature apparently has made very little effort to wall off the infection and prevent its spreading. In treating destroyed apical areas, the general physical condition of the patient should always be a consideration.

In the other type, where the destroyed area is surrounded by a line of condensed bone which seems to limit the infected area, apparently nature has attempted to wall off this diseased area and a condition very similar to that shown by Sir Kenneth Goadby in his *Bone Studies of Latent War Wounds* is apparent. Sir Kenneth Goadby calls these areas "loculi." Areas of this type, where they are not too large, bid fair for treatment if the apical cementum is not absorbed or shows condensation.

After determining that conditions are favorable for treatment, what methods should be used to bring about a condition favorable for restoration of the subapical bone, and may we expect a reproduction of the destroyed area? For a great many years, the treatment of root-canals and periapical diseases has been empirical, and we are all familiar with the old methods of the use of the essential oils, phenol, iodine, etc., and apparently these methods are still in vogue in some localities. The futility of these procedures is too well known to require comment, but there is one thing to which I wish particularly to call your attention and that is to the nostrums which are on the market for the purpose of clearing up these diseased areas. These nostrums are advertised in our dental journals, and that operators are using them to some extent is indicated by the immense sale they have. As the tooth does not give any trouble after the canals are filled with these materials, the dentist believes that the disease has disappeared, but as a matter of fact they are the most dangerous of all materials used in root-canals, for in a majority of such instances the substance used has pernicious power to reduce the vitality of the apical cementum as well as the periapical peridental membrane, and by lowering their vitality and resistance produces a nidus liable to infection either through the blood stream or by the lymphatics, as has been shown by Noyes.

The use of any medicated material in filling root-canals is both unwise and unnecessary. If the material used has sufficient germicidal power to destroy bacteria, it will also have sufficient power to lower the vital resistance of the apical area. It has been my observation that any substance which contains formaldehyde gas is a dangerous substance to use in a root-canal.

About four years ago, it was decided in our office to adopt a definite technic in all root-canal work. This technic will be shown by my associate, Dr. Harry B. Johnston. It is based on a series of experiments I made with egg albumen and the method used in electro-medication, or so-called ionization, combined with the proper root-canal technic and root-canal filling. The experiments which I allude to were made with the idea of finding just exactly how much electric current was required to coagulate albumen. They were made in the following manner:

A hole was bored in the bottom of a test tube and a small platinum wire inserted and sealed in place. The tube was then filled with egg albumen within about one-half inch of the top. In this albumen was imbedded a tooth the root-canal of which had been opened and through which a platinum wire had been passed. Electric wires were then attached to these terminals and the millimeter was carefully watched as the current went through the tooth into the albumen. It was found that anything over a half milliampere would produce immediate coagulation of the albumen and a destruction of this substance in the area of the apex. Therefore it was determined that the amount of current we should use should not exceed a half milliampere. In fact, we seldom go that high.

It should be readily understood that if the area beyond the apex was coagulated or cooked, the active passage of any medicine used in ionization was greatly limited, and the penetration of medicine into the subapical tissues was prevented. If low milliamperage is used, the ions are carried into the tissues. For a long time we used three per cent. chloride of zinc with a pure zinc point, the electric current being applied for about fifteen minutes, but this technic has been changed, as will be shown by Dr. Johnston.

The idea of the electric current producing coagulation and preventing the further penetration of medicine was given me a great many years ago by Dr. F. T. VanWoert of Brooklyn, now of New York City.

The results of these experiments have been carefully checked and will be shown you by Dr. Johnston in his slides. I wish to observe that in the pictures Dr. Johnston will show there is an apparent reproduction of the lamina dura, the rebuilt bone clearly shows trabeculae, and the whole area is not sclerotic but apparently normal.

4. In what length of time should we expect bone restoration?

The length of time required to rebuild the destroyed area around apical root-ends is from four to twelve months, this depending very largely on the amount of area destroyed.

5. Are teeth so treated no longer a menace to health?

So far as we have been able to judge, after four years of experiment, teeth that have been so treated, which show a bone restoration of the character of which we are speaking, have not produced any injury to the patient. In fact, where we have cleared the mouth of other teeth which have been more seriously diseased and have extracted these teeth, there has been no return of the symptoms from which the patient was suffering.

While on the subject of diagnosis, I wish to call your attention to a few pictures of cases of pyorrhea which will definitely show extreme bone condition, and these pictures are used as a means of diagnosis before any treatment is indicated. See *Journal N. D. A.*, February, 1921.

Where the bone shows disease of the rarefying type, it is seldom wise to attempt any treatment, but where the bone shows a condensing line the cases usually yield readily to treatment. It is surprising to note what a small amount of bone will hold a tooth firmly in position if the surrounding area is of the condensing type. Many cases of pyorrhea that do not get well are of the rarefying type, and where the bone is infiltrated treatment usually fails.

I do not claim anything for this method but only show the results of four years of constant work. During this time we have not varied our technique or treatment except in experimental cases. Our results have been rather satisfactory with our methods. All my pictures were taken with the gas tube. Thus far we have not been able to get tubes of other types to do as satisfactory work for us. We use a seven-inch tube, a four-inch spark back-up, twenty-five milliamperes per current, four to five seconds exposure.—*Journal N. D. A.*, February, 1921.

